# Implementing Prenatal Diagnosis Based on Cell-Free Fetal DNA: Accurate Identification of Factors Affecting Fetal DNA Yield

**DOI:** 10.1371/journal.pone.0025202

**Published:** 2011-10-04

**Authors:** Angela N. Barrett, Bernhard G. Zimmermann, Darrell Wang, Andrew Holloway, Lyn S. Chitty

**Affiliations:** 1 NE Thames Regional Molecular Genetics Laboratories, Great Ormond Street Hospital for Children, London, United Kingdom; 2 Fluidigm Corporation, South San Francisco, California, United States of America; 3 University College London Institute of Child Health, London, United Kingdom; 4 University College Hospital NHS Foundation Trust, London, United Kingdom; Tor Vergata University of Rome, Italy

## Abstract

**Objective:**

Cell-free fetal DNA is a source of fetal genetic material that can be used for non-invasive prenatal diagnosis. Usually constituting less than 10% of the total cell free DNA in maternal plasma, the majority is maternal in origin. Optimizing conditions for maximizing yield of cell-free fetal DNA will be crucial for effective implementation of testing. We explore factors influencing yield of fetal DNA from maternal blood samples, including assessment of collection tubes containing cell-stabilizing agents, storage temperature, interval to sample processing and DNA extraction method used.

**Methods:**

Microfluidic digital PCR was performed to precisely quantify male (fetal) DNA, total DNA and long DNA fragments (indicative of maternal cellular DNA). Real-time qPCR was used to assay for the presence of male SRY signal in samples.

**Results:**

Total cell-free DNA quantity increased significantly with time in samples stored in K_3_EDTA tubes, but only minimally in cell stabilizing tubes. This increase was solely due to the presence of additional long fragment DNA, with no change in quantity of fetal or short DNA, resulting in a significant decrease in proportion of cell-free fetal DNA over time. Storage at 4°C did not prevent these changes.

**Conclusion:**

When samples can be processed within eight hours of blood draw, K_3_EDTA tubes can be used. Prolonged transfer times in K_3_EDTA tubes should be avoided as the proportion of fetal DNA present decreases significantly; in these situations the use of cell stabilising tubes is preferable. The DNA extraction kit used may influence success rate of diagnostic tests.

## Introduction

The presence of cell-free fetal DNA in the maternal circulation offers an alternative source of fetal genetic material for prenatal diagnosis [1]. It can be accessed without the risk of miscarriage associated with the invasive procedures required to obtain chorionic villi or amniocytes [2]. However, there are significant technical challenges associated with this non-invasive approach to prenatal diagnosis, since cell-free fetal DNA in early pregnancy usually constitutes less than 10% of total circulating free DNA [3]. Current clinical applications of non-invasive prenatal diagnosis (NIPD) are restricted to the identification of alleles present in the fetus but not in the mother (either inherited from the father or arising *de novo*), including sex determining genes [4], and *RHD* in RhD negative mothers [5], as well as occasional diagnosis of monogenic disorders [6], such as achondroplasia [7]. Recent publications have described alternative approaches to extend the range of monogenic disorders which may be amenable to prenatal diagnosis based on cffDNA to include X-linked disorders, such as haemophilia [8] as well as recessive ones, for example beta-thalassaemia [9]. However, reports of clinical use of cffDNA for clinical indications consistently describe failure to produce a conclusive result in a small proportion of cases [10]–[12]. NIPD based on cell-free fetal DNA in situations where both parents carry a mutant allele for recessively-inherited monogenic disorders or for the diagnosis of fetal aneuploidy is more challenging and may depend on detection of small changes in relative proportions of alleles using methods such as digital PCR [13], [14] or next generation sequencing [15], [16], [17]. In this context, optimization of the proportion of cell-free fetal DNA yield may become critical. Studies in healthy volunteers have shown that total cell-free DNA is increased in serum compared to plasma samples, and it increases when blood is stored for 24 hours prior to centrifugation, the effects presumed to be secondary to cell lysis over time [18]. Further studies using blood taken from pregnant women showed a similar increase in total cell-free DNA over time, but using real-time PCR demonstrated that the absolute quantity of cell-free fetal DNA remained constant [19].

Genetic diagnostic services in the UK and Europe tend to be regionalized, and current practice is that patient samples are transported to the appropriate laboratory for analysis. Whilst transport time is often less than a day, it can be considerably longer, with some samples taking up to a week to arrive. Here, we evaluate the factors that may maximize the yield of cell-free fetal DNA, using digital PCR to accurately determine quantities of both short (45–46 bp) and long (188–192 bp) amplicons in parallel assays to count the number of short (predominantly fetal) and long DNA (mainly maternal) fragments [20], [21]. These data will inform development of standards needed for implementation of this technology into routine clinical practice.

## Materials and Methods

Blood samples were collected from women attending the Fetal Medicine Unit at University College Hospital NHS Foundation Trust, London, for an invasive diagnostic test. Informed consent was obtained prior to venepuncture and the study was approved by the UCLH Ethics Committee A (ref 01/0095).

This study consists of four modules for testing different conditions of storage prior to centrifugation. In a fifth module we identified cases referred to our laboratory for fetal sex determination because the mother was a carrier of a sex linked disorder, where the test was inconclusive or had given a discordant result. In these cases, where there was sufficient plasma stored we used a different extraction kit before performing the PCR assay.

### Module 1

DNA was extracted from 52 banked plasma samples [22] processed at variable, but known, time intervals (range 2–24 hours) following blood draw into K_3_EDTA collection tubes. Plasma was stored at −80°C and samples were processed at one time-point only in this module.

### Module 2

To investigate the effect of varying the time interval between blood draw and sample processing within individuals, three 10 ml K_3_EDTA tubes of blood were collected from twelve pregnant women. One tube was processed immediately (0 hour sample), and the other two were stored at 4°C for four or 24 hours before processing.

### Module 3

To compare the effects of storage temperature on the composition of the cell-free DNA, six 5 ml K_3_EDTA tubes of blood were collected from ten pregnant women. Tube 1 was processed immediately (0 hour sample). Tubes 2 and 3 were stored for eight hours, one at room temperature and the second at 4°C. Tubes 4 and 5 were kept for 24 hours at room temperature and 4°C respectively. The 6th tube was stored at room temperature for 72 hours and then processed.

### Module 4

To evaluate the use of the cell-stabilizing tubes (Cell-free DNA BCTs, Streck™) in comparison to K_3_EDTA tubes, blood from 20 individuals was drawn into three of each tube type. One K_3_EDTA and one cell-stabilizing tube was processed immediately (0 hour sample), one of each at 24 hours, and one of each at 72 hours. All samples were stored at room temperature.

### Module 5

To explore the possibility of reducing the number of inconclusive results following NIPD for fetal sex determination the Qiagen QIAamp Circulating Nucleic Acid (CNA) kit was used to extract DNA from frozen plasma samples previously found to be inconclusive when extracted using the QIAamp MinElute Virus Spin™ (QV). Quantitative real time PCR using a Y-chromosome specific assay (*SRY*) was carried out on each sample extracted in parallel, using CCR5 as a control to confirm the presence of DNA in the extracted sample.

For all modules plasma was separated from the blood cells by centrifugation at 1500× g for ten minutes. The supernatant was then transferred to fresh tubes, ensuring that the buffy coat remained intact. The plasma was centrifuged at 16000× g for ten minutes to remove any remaining cells, transferred into two ml Lo-Bind tubes (Eppendorf UK) and stored at −80°C until DNA extraction.

DNA was extracted from 2 ml of plasma using the QIAamp Circulating Nucleic Acid kit (Qiagen, UK) according to manufacturer's instructions, and was eluted into a final volume of 50 µl elution buffer.

Two duplex digital PCR assays were established for the simultaneous amplification of either short or long sequences from the Y chromosome (*DYS14*) and chromosome 18 (*ZCCHC2*). Sequences of primers and probes are listed in Table 1. *ZCCHC2* probes were labeled with Cal Fluor Orange and *DYS14* probes were labeled with FAM (Biosearch Technologies, Novato, CA). The short (45–46 bp) assay was used to determine the total quantity of DNA (maternal plus fetal) and the percentage of male fetal DNA in a plasma DNA sample. The long (188–192) bp assay was used to quantify the number and percentage of long DNA molecules (predominantly maternal).

**Table 1:** Primer and probe sequences for the digital PCR assays.

	Name	Sequence
Short Duplex	ZCCHC2-F	TACCTGCGCTGTGGCCAATCGAATAAAACACACAGTACCGCGGCAGAG
	ZCCHC2-46R	CAGCACTGATGTAAGAGGTGCTG
	TQ1 Probe	CaO-ATTCGATTGGCCACAGCGCAGGTA-DQ
	DYS_F_TQ2	AAGCTCAGTCATTTCCAGGTGTGCGAAAAGGGCCAATGTTGTATCCTTCTC
	DYS_R 045	ACTAGAAAGGCCGAAGAAACACT
	TQ2 Probe	FAM-TCGCACACCTGGAAATGACTGAGCTT-DQ
Long Duplex	ZCCHC2_F	ACACACAGTACCGCGCAGAG
	ZCCHC2_194R	GGTCCAGGCATTGGATTAGGAT
	ZCCHC2 PB	CaO-CAGCACCTCTTACATCAGTGCTGTGG-DQ
	DYS_F	GGGCCAATGTTGTATCCTTCTC
	DYS_188R	CGCATGCAGGACAATAGTACCC
	DYS14 PB	FAM-TGTTTCTTCGGCCTTTCTAGTGGAGAGG-DQ

Digital PCR was carried out using 12.765 Digital Array™ chips on the BioMark™ System (Fluidigm, San Francisco). The chips contain 12 panels with 765 chambers of 6 nL volume. Six samples were analysed per chip, using one panel each for the short assay duplex and one each for the long assay duplex. A final concentration of 900 nM of each primer and 200 nM of each probe was used with TaqMan Gene Expression Master Mix (Applied Biosystems). The following PCR conditions were used: 50°C for two minutes, 95°C for ten minutes, then 45 cycles of 20 s at 95°C and one minute annealing/extension at 60°C. Fluorescent signals were read each cycle at 95°C.

The number of target molecules per panel was determined using BioMark Digital PCR Analysis software. These counts were then converted into copies per ml of plasma. Since the *DYS14* assays detect 25–30 copies per Y chromosome, 30 copies per Y chromosome was factored into converting the number of DYS14 targets to the number of Y targets. Paired comparisons of samples from the same individuals were performed, thereby avoiding distortion of results due to any variation in copy number of *DYS14* between individuals.

Real-time quantitative PCR was carried out using the ABI7300 system (Applied Biosystems). An *SRY* Taqman assay (Applied Biosystems) was used to detect male DNA, and a *CCR5* Taqman assay was used as a control to detect total DNA. 25 µl reactions were prepared using 1× Taqman assay, 1× Taqman Universal PCR Mastermix with no UNG (Applied Biosystems), and 5 µl of plasma DNA. Six replicates were performed per sample for *SRY* detection and three replicates for CCR5 per extract. Standard curves were constructed for each gene using male genomic DNA (Promega).

Reporting criteria for fetal sexing at the North East Thames Regional Molecular Genetics laboratory are summarized in Table 2. Two separate maternal samples are analyzed; these can be taken 1 week apart or at the same time after 9 weeks gestation.

**Table 2:** Reporting criteria for fetal sexing.

No. of replicates of SRY<Ct45	Sex assignment
0	Female
1	Female if Ct>40;
2–4	Inconclusive
5,6	Male (standard deviation should be no more than 1.5)

Data analysis was performed using Sigma Stat 17.0 (SPSS Inc, Chicago, IL). Significance was determined using a paired Student's t-test and P<0.05 was considered statistically significant.

## Results

### Module 1

**Figure 1:**
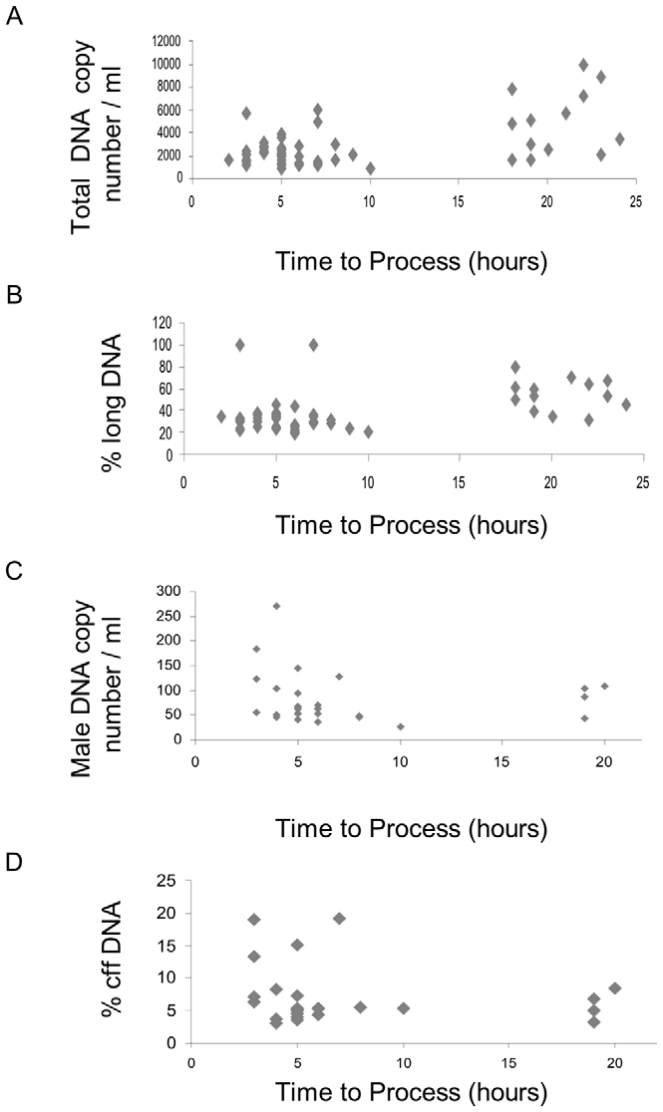
Scatter plots showing (A) the total DNA copy number per ml of plasma, (B) the percentage of long DNA, (C) the male DNA copy number per ml of plasma, and (D) the percentage of fetal DNA in plasma processed at different time points from blood samples collected into EDTA tubes.

Analysis of plasma samples, median gestational age 13+0 weeks (Interquartile range (IQR) 12+4 to 13+4 weeks), processed at known intervals following blood draw, showed that there is an increase of both total DNA (Fig. 1A) and the percentage of long DNA fragments (Fig. 1B) as time to process increases. There was no significant change in the number of copies of male DNA (Fig. 1C), and thus there is a gradual decrease in the proportion of cell-free fetal DNA over time (Fig. 1D). For raw data for all modules, see supporting information Table S1.

### Module 2

**Figure 2:**
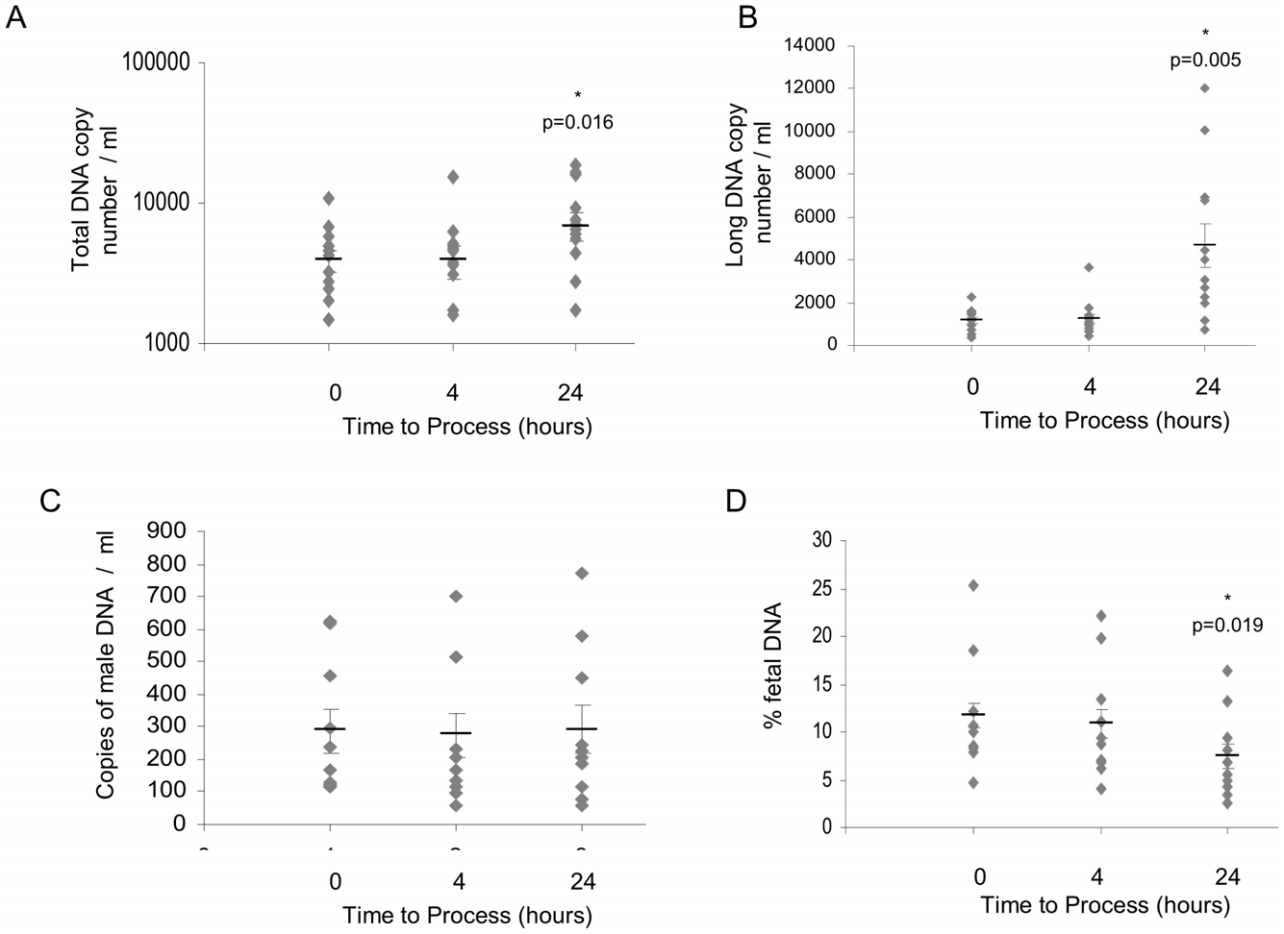
Samples processed at 0, 4, and 24 hours, stored at 4°C. (A) Total DNA copy number per ml of plasma, (B) long DNA copy number per ml of plasma; n = 12. (C) Male DNA copy number per ml of plasma, and (D) percentage of fetal DNA; n = 10. Mean for each set of data is represented by a black bar, SEM is indicated, *indicates statistical significance.

No significant difference was seen between the total DNA or long DNA copy number per ml of plasma at 0 and 4 hours storage of blood at 4°C (Figs. 2A and 2B). Between 0 and 24 hours the median copy number of total or long DNA copy number increases (Figs. 2A and 2B); however, no difference was detected in the median copy numbers of fetal DNA at any time-point (147 copies/ml (0 hours), 151 copies/ml (4 hours) and 196 copies/ml (24 hours); Fig. 2C). The percentage of the cell-free fetal DNA remains the same after four hours, but decreases between 0 and 24 hours (Fig. 2D). The median gestational age of the fetuses from women studied in this module was 16+2 weeks (interquartile range (IQR) 13+2 to 20+6 weeks). For summary statistics for Module 2, and for subsequent modules, see supporting information Table S2.

### Module 3

**Figure 3:**
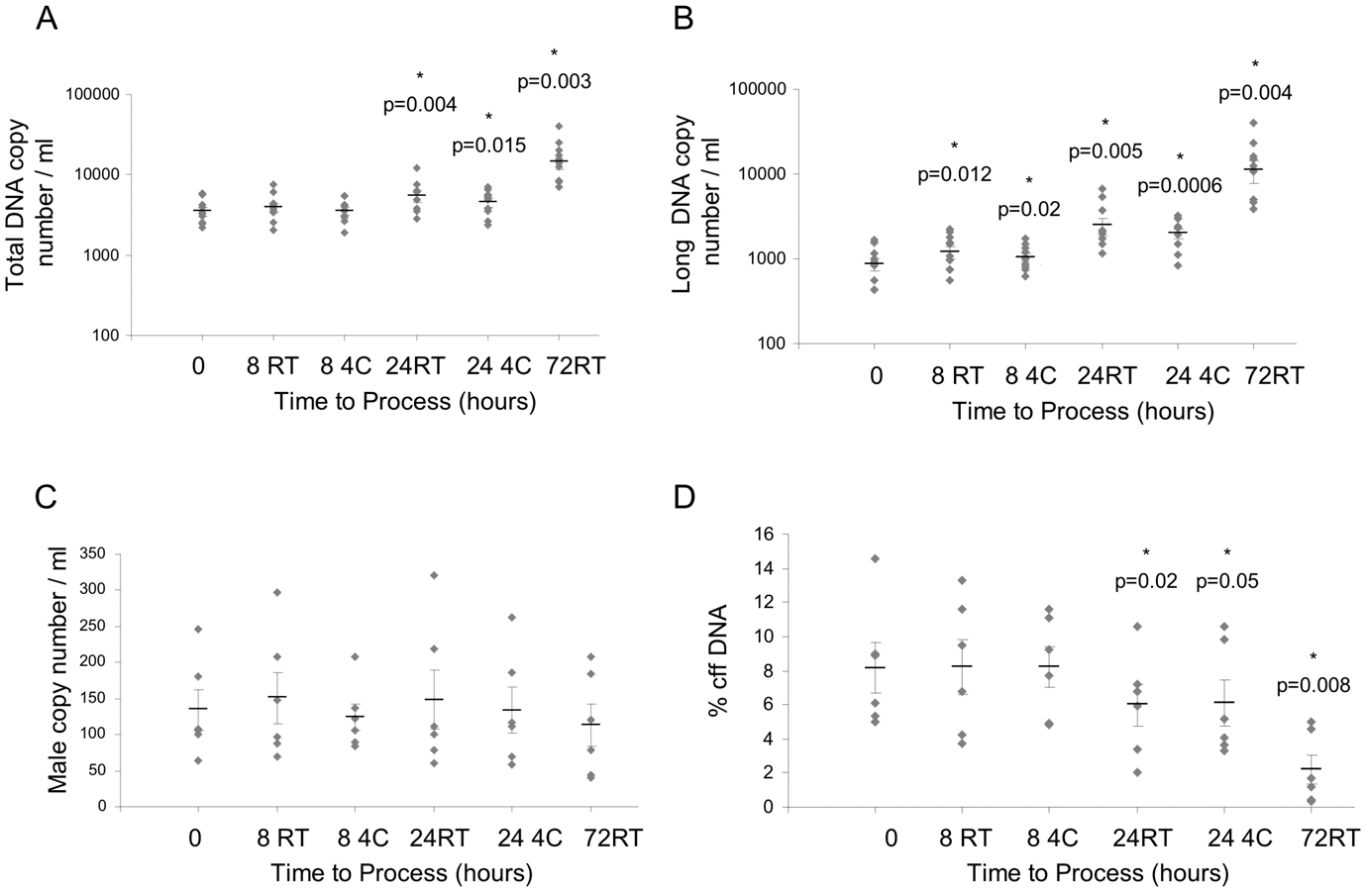
Effects of time and storage temperature on cell-free DNA quantities. Plasma was processed at 0, 8, 24 and 72 hours, and stored at room temperature (RT) or 4°C (4C). (A) Total DNA copy number per ml of plasma, (B) long DNA copy number per ml of plasma; n = 10. (C) Male DNA copy number per ml of plasma, and (D) percentage of fetal DNA; n = 6. *indicates statistical significance.

Storage of blood at 4°C had no effect on the change over time in level of total or long DNA copy number compared with storage at room temperature (Figs. 3A and 3B). As in Module 2, no change was observed in total copies of male DNA (Fig. 3C), resulting in a decrease in the percentage of fetal DNA with time (Fig. 3D) regardless of storage temperature. The median gestational age of pregnancies tested in this module was 14+4 weeks (IQR 13+4 to 19+3 weeks).

### Module 4

**Figure 4:**
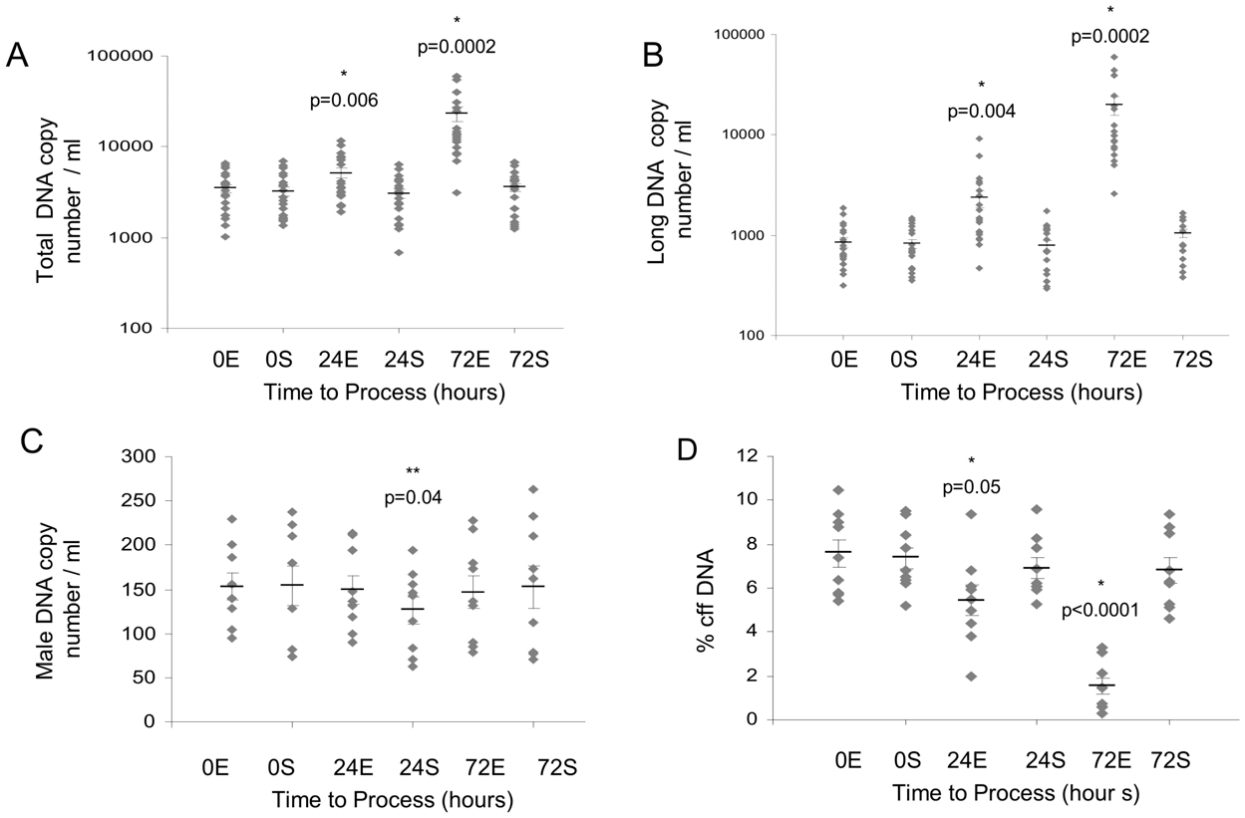
Samples were stored for 0, 24 or 72 hours in either K_3_EDTA tubes (E) or cell-stabilizing tubes (S). (A) Total DNA copy number per ml of plasma, (B) long DNA copy number per ml of plasma; n = 20. (C) Male DNA copy number per ml of plasma, and (D) percentage of fetal DNA; n = 9. *indicates statistical significance between samples in K_3_EDTA tubes; **indicates significance between the cell-stabilizing tubes.

In this module samples from 20 pregnant women, nine of whom were carrying male fetuses (median gestation 14+4 weeks, IQR 12+6 to 16+1 weeks) were examined. As previously noted in plasma from blood collected into K_3_EDTA tubes, there was a significant increase in total DNA copy number at both 24 hours and 72 hours (Fig. 4A) with an associated decrease in proportion of fetal DNA. However, when the blood was collected and stored in cell-stabilizing tubes no change in the median quantity of total DNA was observed at 24 hours, and there was only a slight increase at 72 hours. The same absolute increase in copies was seen with the K_3_EDTA tubes for long DNA (Fig. 4B), and again, there was no increase in copy number with the cell-stabilizing tubes at 24 hours. After 72 hours there was a small but clearly significant increase in long copy number. There was a small but significant decrease in the total quantity of male DNA in plasma from samples stored in the cell-stabilizing tubes for 24 hours, but this decrease was not seen in the samples stored for 72 hours, nor would it be significant at the 99% confidence level. There was no decrease in the quantity of male DNA in samples collected into K_3_EDTA tubes at any time point (Fig. 4C). Consequently, whilst the percentage of fetal DNA decreased significantly in samples stored for 24 and 72 hours in K_3_EDTA tubes, there was no significant difference in the percentage of cell-free fetal DNA in samples stored for three days in cell-stabilizing tubes (Fig. 4D).

### Module 5

DNA from fourteen pregnancies previously reported to be inconclusive (including two pregnancies in which two samples were taken a week apart and were both reported to be inconclusive) were re-examined using real time PCR. Twelve samples that were reported as inconclusive using the QV kit were identified as male using DNA extracted using the CNA kit, and the result obtained matched the birth outcome for nine of these (three others have not been born yet) (table 3). Two samples were still inconclusive, but a repeat of the test using the same sample may have been enough to give a definite result.

**Table 3:** Reduction in the number of inconclusive results obtained using the Qiagen CNA kit compared to the Qiagen QV kit.

Sample	Gestation	No. of SRY Positive (QV)	No. of SRY Positive (CNA)	Average SRY (CNA kit)	Average CCR5 (CNA kit)	Predicted Outcome (CNA kit)	Birth Outcome
1	8	3/6	5/6	35	25	Male	Male
2	8+6	3/6	5/6	35	28	Male	Male
3	17	3/6	6/6	34	30	Male	Male
4	7+6	0/6	3/6	35	27	Inconclusive	Male
5	8+4	0/6	6/6	37	27	Male	Male
6	6+3	0/6	6/6	34	28	Male	Male
7	9+1	3/6	6/6	35	28	Male	Male
8	-	3/6	6/6	34	27	Male	Male
9	7+2	4/6	6/6	35	25	Male	Male
10	7	1/6	6/6	36	26	Male	Male
11	11+	0/6	6/6	35	28	Male	-
12	9	2/6	6/6	35	28	Male	-
13	7	0/6	1/6	-	27	Inconclusive	-
13b	8	2/6	5/6	35	27	Male	-
14	8+3	3/6	6/6	35	27	Male	-
14b	9+3	3/6	6/6	36	27	Male	-

## Discussion

In this study we have shown that the increase in total cell free DNA that occurs with increasing time from blood draw can largely be prevented by taking blood into tubes which stabilize maternal cells, thus allowing the proportion of cell-free fetal DNA to remain more or less constant. Previous studies have all used quantitative real-time PCR to quantify cell-free DNA in samples under different conditions. Here, we apply digital PCR technology to more accurately define the copy numbers of total DNA using very short PCR assays, and use longer PCR amplicons (detecting mainly maternal cell-free DNA) to determine the effect of various pre-analytical factors on yield and composition of cell-free DNA. Digital PCR has an advantage over quantitative PCR since it is able to discriminate differences of less than twofold with great accuracy. Since the technology is based on counting the template molecules, there is no need to normalize to a reference, removing any effect secondary to differences in efficiency in the PCR reactions for two genes or between samples and a reference.

A short PCR assay for the *ZCCHC2* gene on chromosome 18 was used to calculate the total copy number of cell-free DNA per ml of plasma, whilst a long assay for the same gene was used to determine the contribution of maternal cellular DNA to this figure. The short *DYS14* assay indicated the total quantity of fetal DNA in the sample; only approximately 10% of the male DNA was detected with the long assay for *DYS14*, confirming that fetal DNA is predominantly apoptotic. The absolute copy number increases after prolonged storage were the same as measuring with the short PCR assays, indicating that newly released cellular DNA is exclusively long and that no fragmentation of the cell-free DNA occurs after the blood draw.

In the first series of experiments we analyzed individual blood samples prepared at different time-points after blood draw and demonstrated that there is a significant increase in total DNA, but not cell-free fetal DNA as the time to process increases. These samples were not only prepared at varying time intervals, they were also taken at gestational ages across pregnancy. It is known that cell-free fetal DNA levels increase with gestation [3] and that maternal cell-free DNA can fluctuate on a daily basis [23]. To eliminate any possible bias due to these, further experiments were performed to assess the effect of time to process within women, storage temperature, and cell-stabilizing tubes by taking multiple samples from the same individual at the same time. The decrease in proportion of cell-free fetal DNA was only prevented or reduced by taking blood into tubes which stabilized maternal blood cells; gestation and storage temperature had no effect. We have demonstrated that this is not a result of degradation as the fetal DNA levels remain constant; rather additional maternal DNA is released, and this DNA is predominantly (if not exclusively) long. The percentage of long DNA (see supporting information in Tables S1 and S2) can be used to determine the quality of a blood plasma sample for further analysis. The lower the percentage of long DNA, the better, since this indicates that there is less contamination from maternal cells of the cell-free DNA. Previous studies, using real time PCR suggested that cell-stabilizing tubes could maintain the proportion of cell-free fetal DNA over long periods [24]. However, using the more sensitive method of digital PCR, we have shown that total cell-free DNA does increase gradually over time, such that by 72 hours there was a small and statistically very significant increase of long total, and thus maternal, DNA. This observation may have implications for clinical application and it would be interesting to see if this effect is more pronounced with longer time intervals. Of note, the decrease in proportion of cell-free fetal DNA in the small numbers of EDTA samples posted to the laboratory did not appear to affect SRY real-time PCR assay. However, if there is significant delay in sample transfer this might affect performance, as suggested by Finning et al where they attributed two of their three false RhD-negative results of fetal RhD typing to postal delays of up to two weeks [25]. Furthermore, it is likely that prompt preparation of plasma or the use of collection tubes that prevent lysis of maternal cells, will be required to optimize the proportion of cell-free fetal DNA present when doing analyses based on sensitive counting of copy numbers for the estimation of allelic ratios.

Our analyses may help to shed some light on a controversial report by Dhallan, who reported increased recovery of fetal DNA to a median of 25% from blood collected into formaldehyde-containing tubes [26]. Whilst some groups were able to replicate these findings [27], others found the differences in percentage of fetal DNA to be insignificant [28], [29]. It has been suggested that these contradictory findings were caused by different processing times between the untreated samples and the formaldehyde stabilization samples that were being compared [30]. We have shown that at four hours and eight hours following blood draw there is no difference in proportion of fetal DNA in plasma from cells stored in K_3_EDTA tubes or cell-stabilization tubes, but after 24 hours' storage there are significant benefits to using cell-stabilization tubes. Another discrepancy is that Dhallan detected a much greater percentage of fetal DNA using a different technology to others using real-time PCR. The percentage of cell-free fetal DNA in the maternal plasma sample was determined by PCR using serially-diluted plasma DNA and observing the highest serial dilution in which the gene was detected. This limiting dilution approach can be viewed as a precursor to microfluidic digital PCR which, by using hundreds of replicates at a limiting dilution and poisson statistics, enables a much more precise measurement of DNA. The number of fetal copies detected by Dhallan but not the proportion fits very well with our results. It is most probable that the reported median 25% fetal DNA was actually a result of under-detection of the total DNA control.

Finally we have shown that the use of the QIAamp Circulating Nucleic Acid™ kit for cffDNA extraction has potential to decrease the number of inconclusive results and increase accuracy by avoiding false-negative results in maternal samples used to determine fetal sex in pregnancies at risk of sex linked disorders. This may be a reflection of the increased volume of plasma used for the extraction using this kit (2 ml) as compared with the Qiagen QIAamp MinElute Virus Spin™ (QV) which uses 400 µl. However, due to the small volumes of plasma available for analysis in this module we were unable to accurately evaluate the quantity of cffDNA present. These results indicate the need for further evaluation of DNA extraction kits to optimise the quantity of cffDNA available for definitive diagnostic analysis.

In summary, we have shown that storage of blood samples at 4°C provides no advantage over storage at room temperature for maximizing the proportion of cell-free fetal DNA in the plasma. If possible, immediate centrifugation of a sample after blood draw is preferable; when time to processing is going to be greater than eight hours, the use of tubes containing material to stabilize cells is useful to optimize cell-free fetal DNA levels. The DNA extraction kit, and/or volume of plasma extracted may influence reliability of testing in the clinical situation. Our findings may have significant implications for implementing non-invasive prenatal diagnosis based on cell-free fetal DNA into clinical practice.

## Supporting Information

Table S1 **Raw data.**
(DOC)

Table S2 **Summary statistics.**
(DOC)
