# Supplementary material for: Implementing Prenatal Diagnosis Based on Cell-Free Fetal DNA: Accurate Identification of Factors Affecting Fetal DNA Yield
Source: PLoS One. 2011 Oct 4;6(10):e25202. doi: 10.1371/journal.pone.0025202 (PMC3187716; doi:10.1371/journal.pone.0025202)
Supplement: Table S1 — Raw data. (DOC) [file pone.0025202.s001.doc]

**Supplementary Data** **Table 1:** Raw Data

|  | **Sample No.** | **Fetal Age (Weeks)** | **Time to Process (hours)** | | **Total DNA** | **Long**  **DNA** | **Total Male DNA** | **% long DNA** | **Fetal DNA (%)** |
| --- | --- | --- | --- | --- | --- | --- | --- | --- | --- |
| **Module 1** | 1 | 12+2 | 10 | 960 | | 202 | 26 | 21 | 5.4 |
|  | 2 | 12+6 | 6 | 1328 | | 279 | 36 | 21 | 5.4 |
|  | 3 | 13+5 | 5 | 2230 | | 781 | 40 | 35 | 3.5 |
|  | 4 | 13+0 | 19 | 1759 | | 686 | 44 | 39 | 5.0 |
|  | 5 | 13+2 | 4 | 2895 | | 984 | 46 | 34 | 3.1 |
|  | 6 | 13+5 | 8 | 1763 | | 511 | 49 | 29 | 5.5 |
|  | 7 | 12+6 | 4 | 2719 | | 1033 | 50 | 38 | 3.7 |
|  | 8 | 13+4 | 5 | 2632 | | 1211 | 53 | 46 | 4.0 |
|  | 9 | 13+4 | 6 | 1987 | | 517 | 54 | 26 | 5.4 |
|  | 10 | 13+5 | 5 | 2052 | | 780 | 54 | 38 | 5.3 |
|  | 11 | 12+4 | 3 | 1573 | | 535 | 56 | 34 | 7.2 |
|  | 12 | 12+3 | 5 | 2751 | | 908 | 63 | 33 | 4.6 |
|  | 13 | 12+0 | 6 | 2901 | | 638 | 64 | 22 | 4.4 |
|  | 14 | 13+0 | 5 | 2591 | | 933 | 64 | 36 | 5.0 |
|  | 15 | 13+0 | 5 | 884 | | 212 | 67 | 24 | 15.2 |
|  | 16 | 12+3 | 19 | 5234 | | 3140 | 86 | 60 | 3.3 |
|  | 17 | 13+6 | 5 | 3678 | | 883 | 95 | 24 | 5.1 |
|  | 18 | 13+0 | 4 | 2488 | | 921 | 103 | 37 | 8.3 |
|  | 19 | 13+4 | 19 | 3082 | | 1664 | 104 | 54 | 6.8 |
|  | 20 | 13+4 | 20 | 2553 | | 894 | 109 | 35 | 8.5 |
|  | 21 | 13+1 | 3 | 1299 | | 299 | 123 | 23 | 18.9 |
|  | 22 | 13+2 | 7 | 1340 | | 415 | 128 | 31 | 19.1 |
|  | 23 | 12+5 | 5 | 3935 | | 944 | 145 | 24 | 7.4 |
|  | 24 | 13+0 | 3 | 2467 | | 2467 | 163 | 100 | 13.2 |
|  | 25 | 12+4 | 3 | 5779 | | 1849 | 184 | 32 | 6.4 |
|  | 26 | 14+4 | 4 | 3262 | | 848 | 272 | 26 | 16.7 |
|  | 27* | 13+3 | 8 | 3042 | | 973 | 1690 | 32 | 111.1 |
|  | 28 | 12+3 | 2 | 1651 | | 578 | 0 | 35 | 0.0 |
|  | 29 | 12+4 | 5 | 1740 | | 679 | 0 | 39 | 0.0 |
|  | 30 | 12+5 | 7 | 1560 | | 468 | 0 | 30 | 0.0 |
|  | 31 | 12+4 | 24 | 3447 | | 1586 | 0 | 46 | 0.0 |
|  | 32 | 13+1 | 23 | 9012 | | 6128 | 0 | 68 | 0.0 |
|  | 33 | 13+2 | 6 | 1342 | | 268 | 0 | 20 | 0.0 |
|  | 34 | 13+1 | 23 | 2078 | | 1122 | 0 | 54 | 0.0 |
|  | 35 | 13+0 | 5 | 1197 | | 407 | 0 | 34 | 0.0 |
|  | 36 | 14+3 | 18 | 1644 | | 1003 | 0 | 61 | 0.0 |
|  | 37 | 11+5 | 18 | 7930 | | 6344 | 0 | 80 | 0.0 |
|  | 38 | 14+5 | 9 | 2128 | | 511 | 0 | 24 | 0.0 |
|  | 39 | 12+1 | 7 | 5005 | | 5005 | 0 | 100 | 0.0 |
|  | 40 | 12+4 | 6 | 1290 | | 568 | 0 | 44 | 0.0 |
|  | 41 | 12+2 | 21 | 5751 | | 4083 | 0 | 71 | 0.0 |
|  | 42 | 13+4 | 22 | 9926 | | 3176 | 0 | 32 | 0.0 |
|  | 43 | 11+2 | 18 | 4899 | | 2498 | 0 | 51 | 0.0 |
|  | 44 | 13+2 | 3 | 1702 | | 528 | 0 | 31 | 0.0 |
|  | 45 | 13+6 | 6 | 1328 | | 372 | 0 | 28 | 0.0 |
|  | 46 | 13+4 | 4 | 2320 | | 719 | 0 | 31 | 0.0 |
|  | 47 | 13+1 | 22 | 7335 | | 4694 | 0 | 64 | 0.0 |
|  | 48 | 12+4 | 3 | 2127 | | 532 | 0 | 25 | 0.0 |
|  | 49 | 13+6 | 5 | 2166 | | 563 | 0 | 26 | 0.0 |
|  | 50 | 12+5 | 5 | 1392 | | 501 | 0 | 36 | 0.0 |
|  | 51 | 13+5 | 7 | 6136 | | 2270 | 0 | 37 | 0.0 |
|  | 52 | 13+1 | 7 | 1289 | | 464 | 0 | 36 | 0.0 |
| **Module 2** | 1 | 32+3 | 0 | 6655 | | 1464 | 619 | 22 | 18.6 |
|  |  |  | 4 | 5219 | | 1357 | 516 | 26 | 19.8 |
|  |  |  | 24 | 7098 | | 4117 | 577 | 58 | 16.3 |
|  | 2 | 17+5 | 0 | 4986 | | 1595 | 117 | 32 | 4.7 |
|  |  |  | 4 | 4784 | | 1387 | 94 | 29 | 4.0 |
|  |  |  | 24 | 4388 | | 2282 | 77 | 52 | 3.5 |
|  | 3 | 19+3 | 0 | 2780 | | 753 | 116 | 27 | 8.4 |
|  |  |  | 4 | 1608 | | 637 | 56 | 40 | 7.0 |
|  |  |  | 24 | 2749 | | 1157 | 58 | 42 | 4.2 |
|  | 4 | 13+6 | 0 | 5871 | | 1553 | 293 | 26 | 10.0 |
|  |  |  | 4 | 3766 | | 1087 | 165 | 29 | 8.8 |
|  |  |  | 24 | 5568 | | 2004 | 226 | 36 | 8.1 |
|  | 5 | 12+4 | 0 | 2423 | | 520 | 128 | 21 | 10.6 |
|  |  |  | 4 | 3650 | | 831 | 203 | 23 | 11.1 |
|  |  |  | 24 | 6019 | | 2695 | 206 | 45 | 6.8 |
|  | 6 | 20+6 | 0 | 10639 | | 2283 | 456 | 21 | 8.6 |
|  |  |  | 4 | 15268 | | 3619 | 516 | 24 | 6.8 |
|  |  |  | 24 | 16145 | | 6749 | 451 | 42 | 5.6 |
|  | 7 | 16+2 | 0 | 4496 | | 1180 | 237 | 26 | 10.6 |
|  |  |  | 4 | 5017 | | 1258 | 232 | 25 | 9.3 |
|  |  |  | 24 | 18762 | | 11998 | 244 | 64 | 2.6 |
|  | 8 | 27+0 | 0 | 4912 | | 1276 | 624 | 26 | 25.4 |
|  |  |  | 4 | 6330 | | 1779 | 698 | 28 | 22.1 |
|  |  |  | 24 | 16359 | | 10030 | 770 | 61 | 9.4 |
|  | 9 | 13+0 | 0 | 4293 | | 1483 | 167 | 35 | 7.8 |
|  |  |  | 4 | 4525 | | 941 | 137 | 21 | 6.1 |
|  |  |  | 24 | 7696 | | 4022 | 186 | 52 | 4.8 |
|  | 10 | 13+6 | 0 | 1476 | | 334 | 0 | 23 | 0.0 |
|  |  |  | 4 | 1747 | | 458 | 0 | 26 | 0.0 |
|  |  |  | 24 | 9234 | | 6896 | 1 | 75 | 0.0 |
|  | 11 | 13+2 | 0 | 3168 | | 971 | 0 | 31 | 0.0 |
|  |  |  | 4 | 3075 | | 979 | 0 | 32 | 0.0 |
|  |  |  | 24 | 6399 | | 3060 | 0 | 48 | 0.0 |
|  | 12 | 31+1 | 0 | 2035 | | 505 | 124 | 25 | 12.2 |
|  |  |  | 4 | 1747 | | 629 | 118 | 36 | 13.5 |
|  |  |  | 24 | 1732 | | 699 | 114 | 40 | 13.1 |
| **Module 3** | 1 | - | 0 | 4178 | | 932 | 1 | 22 | 0.0 |
|  |  |  | 8 RT | 3464 | | 979 | 0 | 28 | 0.0 |
|  |  |  | 8 4C | 4271 | | 1180 | 1 | 28 | 0.0 |
|  |  |  | 24 RT | 4962 | | 1973 | 1 | 40 | 0.0 |
|  |  |  | 24 4C | 5490 | | 2353 | 1 | 43 | 0.0 |
|  |  |  | 72 | 19982 | | 16028 | 0 | 80 | 0.0 |
|  | 2 | 18+1 | 0 | 2423 | | 435 | 107 | 18 | 8.9 |
|  |  |  | 8 RT | 2539 | | 753 | 148 | 30 | 11.6 |
|  |  |  | 8 4C | 2671 | | 738 | 123 | 28 | 9.2 |
|  |  |  | 24 RT | 3766 | | 2182 | 111 | 58 | 5.9 |
|  |  |  | 24 4C | 5335 | | 3246 | 111 | 61 | 4.1 |
|  |  |  | 72 | 24967 | | 23181 | 41 | 93 | 0.3 |
|  | 3 | 14+4 | 0 | 3052 | | 1017 | 1 | 33 | 0.1 |
|  |  |  | 8 RT | 3401 | | 746 | 2 | 22 | 0.1 |
|  |  |  | 8 4C | 3060 | | 870 | 1 | 28 | 0.0 |
|  |  |  | 24 RT | 5708 | | 1740 | 0 | 42 | 0.0 |
|  |  |  | 24 4C | 4838 | | 2346 | 0 | 36 | 0.0 |
|  |  |  | 72 | 15586 | | 10686 | 0 | 69 | 0.0 |
|  | 4 | 13+1 | 0 | 3386 | | 559 | 247 | 17 | 14.6 |
|  |  |  | 8 RT | 4465 | | 1072 | 297 | 24 | 13.3 |
|  |  |  | 8 4C | 4069 | | 800 | 236 | 20 | 11.6 |
|  |  |  | 24 RT | 6019 | | 2027 | 320 | 34 | 10.6 |
|  |  |  | 24 4C | 4947 | | 1926 | 262 | 39 | 10.6 |
|  |  |  | 72 | 8317 | | 5056 | 207 | 61 | 5.0 |
|  | 5 | 26+3 | 0 | 3976 | | 1646 | 106 | 41 | 5.3 |
|  |  |  | 8 RT | 4163 | | 2058 | 87 | 49 | 4.2 |
|  |  |  | 8 4C | 3611 | | 1514 | 89 | 42 | 4.9 |
|  |  |  | 24 RT | 7634 | | 5428 | 78 | 71 | 2.0 |
|  |  |  | 24 4C | 3813 | | 2353 | 69 | 62 | 3.6 |
|  |  |  | 72 | 14080 | | 12611 | 121 | 90 | 1.7 |
|  | 6 | 20+5 | 0 | 5894 | | 1561 | 181 | 26 | 6.1 |
|  |  |  | 8 RT | 6104 | | 1763 | 207 | 29 | 6.8 |
|  |  |  | 8 4C | 5413 | | 1732 | 207 | 32 | 7.7 |
|  |  |  | 24 RT | 6399 | | 2159 | 218 | 34 | 6.8 |
|  |  |  | 24 4C | 7075 | | 3036 | 186 | 43 | 5.2 |
|  |  |  | 72 | 8038 | | 3844 | 184 | 48 | 4.6 |
|  | 7 | 13+2 | 0 | 5669 | | 1157 | 0 | 20 | 0.0 |
|  |  |  | 8 RT | 7580 | | 2229 | 0 | 29 | 0.0 |
|  |  |  | 8 4C | 5467 | | 1328 | 0 | 24 | 0.0 |
|  |  |  | 24 RT | 11936 | | 6795 | 0 | 57 | 0.0 |
|  |  |  | 24 4C | 6554 | | 2959 | 0 | 45 | 0.0 |
|  |  |  | 72 | 17761 | | 14693 | 1 | 83 | 0.0 |
|  | 8 | - | 0 | 3332 | | 862 | 0 | 26 | 0.0 |
|  |  |  | 8 RT | 4450 | | 1561 | 0 | 35 | 0.0 |
|  |  |  | 8 4C | 2920 | | 1002 | 1 | 34 | 0.1 |
|  |  |  | 24 RT | 6376 | | 3720 | 1 | 58 | 0.0 |
|  |  |  | 24 4C | 2625 | | 1126 | 1 | 43 | 0.0 |
|  |  |  | 72 | 12488 | | 10833 | 1 | 87 | 0.0 |
|  | 9 | 15+2 | 0 | 2539 | | 830 | 63 | 33 | 5.0 |
|  |  |  | 8 RT | 3735 | | 1507 | 70 | 40 | 3.7 |
|  |  |  | 8 4C | 3526 | | 1025 | 84 | 29 | 4.8 |
|  |  |  | 24 RT | 3565 | | 1514 | 61 | 42 | 3.4 |
|  |  |  | 24 4C | 3565 | | 1491 | 59 | 42 | 3.3 |
|  |  |  | 72 | 7152 | | 4698 | 44 | 66 | 1.2 |
|  | 10 | 13+0 | 0 | 2229 | | 427 | 100 | 19 | 9.0 |
|  |  |  | 8 RT | 2050 | | 551 | 97 | 27 | 9.5 |
|  |  |  | 8 4C | 1887 | | 614 | 105 | 33 | 11.1 |
|  |  |  | 24 RT | 2819 | | 1157 | 101 | 41 | 7.2 |
|  |  |  | 24 4C | 2376 | | 823 | 116 | 35 | 9.5 |
|  |  |  | 72 | 39637 | | 39637 | 79 | 100 | 0.4 |
| **Module 4** | 1 | 23+4 | 0 E | 6197 | | 1328 | 230 | 21 | 7.4 |
|  |  |  | 0S | 6174 | | 1406 | 210 | 23 | 6.8 |
|  |  |  | 24E | 7230 | | 1996 | 213 | 28 | 5.9 |
|  |  |  | 24S | 6376 | | 1258 | 194 | 20 | 6.1 |
|  |  |  | 72E | 30551 | | 24470 | 228 | 80 | 1.5 |
|  |  |  | 72S | 6850 | | 1522 | 232 | 22 | 6.8 |
|  | 2 | 13+0 | 0 E | 2959 | | 870 | 94 | 29 | 6.4 |
|  |  |  | 0S | 2819 | | 738 | 74 | 26 | 5.2 |
|  |  |  | 24E | 3580 | | 1064 | 99 | 30 | 5.5 |
|  |  |  | 24S | 2710 | | 683 | 71 | 25 | 5.3 |
|  |  |  | 72E | 11439 | | 9855 | 85 | 86 | 1.5 |
|  |  |  | 72S | 2780 | | 699 | 71 | 25 | 5.1 |
|  | 3 | 13+6 | 0 E | 1740 | | 582 | 1 | 33 | 0.1 |
|  |  |  | 0S | 1576 | | 458 | 14 | 29 | 1.8 |
|  |  |  | 24E | 3495 | | 1794 | 1 | 51 | 0.0 |
|  |  |  | 24S | 2353 | | 691 | 0 | 29 | 0.0 |
|  |  |  | 72E | 16145 | | 10779 | 1 | 67 | 0.0 |
|  |  |  | 72S | 2105 | | 691 | 0 | 33 | 0.0 |
|  | 4 | 15+2 | 0 E | 4038 | | 1111 | 0 | 28 | 0.0 |
|  |  |  | 0S | 3270 | | 1048 | 0 | 28 | 0.0 |
|  |  |  | 24E | 7634 | | 3456 | 0 | 45 | 0.0 |
|  |  |  | 24S | 4388 | | 1033 | 0 | 24 | 0.0 |
|  |  |  | 72E | 26785 | | 19345 | 0 | 72 | 0.0 |
|  |  |  | 72S | 4597 | | 1227 | 0 | 27 | 0.0 |
|  | 5 | 15+2 | 0 E | 1592 | | 513 | 1 | 32 | 0.1 |
|  |  |  | 0S | 1778 | | 691 | 8 | 39 | 0.8 |
|  |  |  | 24E | 2283 | | 800 | 0 | 35 | 0.0 |
|  |  |  | 24S | 2322 | | 691 | 1 | 30 | 0.0 |
|  |  |  | 72E | 8379 | | 6306 | 0 | 75 | 0.0 |
|  |  |  | 72S | 1476 | | 489 | 0 | 33 | 0.0 |
|  | 6 | 24+0 | 0 E | 5832 | | 1639 | 0 | 28 | 0.0 |
|  |  |  | 0S | 7484 | | 1219 | 1 | 25 | 0.0 |
|  |  |  | 24E | 7098 | | 2780 | 0 | 39 | 0.0 |
|  |  |  | 24S | 4271 | | 1111 | 0 | 26 | 0.0 |
|  |  |  | 72E | 14693 | | 9816 | 1 | 67 | 0.0 |
|  |  |  | 72S | 4349 | | 1413 | 0 | 33 | 0.0 |
|  | 7 | 18+1 | 0 E | 3285 | | 645 | 155 | 20 | 9.4 |
|  |  |  | 0S | 3798 | | 800 | 180 | 21 | 9.5 |
|  |  |  | 24E | 3145 | | 932 | 148 | 30 | 9.4 |
|  |  |  | 24S | 3238 | | 691 | 156 | 21 | 9.6 |
|  |  |  | 72E | 8480 | | 5506 | 132 | 65 | 3.1 |
|  |  |  | 72S | 3697 | | 1087 | 173 | 29 | 9.4 |
|  | 8 | 15+5 | 0 E | 6485 | | 1856 | 186 | 29 | 5.7 |
|  |  |  | 0S | 5832 | | 1476 | 180 | 25 | 6.2 |
|  |  |  | 24E | 8511 | | 3238 | 212 | 38 | 5.0 |
|  |  |  | 24S | 5646 | | 1740 | 167 | 31 | 5.9 |
|  |  |  | 72E | 13373 | | 8845 | 218 | 66 | 3.3 |
|  |  |  | 72S | 5203 | | 1522 | 162 | 29 | 6.2 |
|  | 9 | 18+3 | 0 E | 2058 | | 606 | 1 | 29 | 0.1 |
|  |  |  | 0S | 1654 | | 621 | 1 | 38 | 0.1 |
|  |  |  | 24E | 2275 | | 893 | 1 | 39 | 0.0 |
|  |  |  | 24S | 1344 | | 443 | 18 | 33 | 2.7 |
|  |  |  | 72E | 3153 | | 2586 | 0 | 82 | 0.0 |
|  |  |  | 72S | 1406 | | 582 | 0 | 41 | 0.0 |
|  | 10 | 12+2 | 0 E | 1771 | | 450 | 1 | 25 | 0.1 |
|  |  |  | 0S | 1592 | | 474 | 0 | 30 | 0.0 |
|  |  |  | 24E | 2182 | | 1079 | 0 | 49 | 0.0 |
|  |  |  | 24S | 1258 | | 342 | 2 | 27 | 0.3 |
|  |  |  | 72E | 13831 | | 12550 | 0 | 91 | 0.0 |
|  |  |  | 72S | 1243 | | 427 | 0 | 34 | 0.0 |
|  | 11 | 12+5 | 0 E | 4834 | | 792 | 139 | 16 | 5.8 |
|  |  |  | 0S | 6873 | | 1398 | 223 | 20 | 6.5 |
|  |  |  | 24E | 3879 | | 1025 | 132 | 26 | 6.8 |
|  |  |  | 24S | 4776 | | 1235 | 146 | 26 | 6.1 |
|  |  |  | 72E | 54074 | | 44079 | 173 | 82 | 0.6 |
|  |  |  | 72S | 6675 | | 1398 | 210 | 21 | 6.3 |
|  | 12 | 15+2 | 0 E | 5114 | | 1258 | 139 | 25 | 5.4 |
|  |  |  | 0S | 4031 | | 1316 | 129 | 33 | 6.4 |
|  |  |  | 24E | 6314 | | 2470 | 137 | 39 | 4.4 |
|  |  |  | 24S | 3681 | | 1165 | 114 | 32 | 6.2 |
|  |  |  | 72E | 12814 | | 8469 | 136 | 66 | 2.1 |
|  |  |  | 72S | 4217 | | 1677 | 112 | 40 | 5.3 |
|  | 13 | 12+0 | 0 E | 3634 | | 629 | 2 | 17 | 0.1 |
|  |  |  | 0S | 2516 | | 734 | 1 | 29 | 0.1 |
|  |  |  | 24E | 3192 | | 1340 | 0 | 42 | 0.0 |
|  |  |  | 24S | 2097 | | 408 | 1 | 19 | 0.1 |
|  |  |  | 72E | 9797 | | 7234 | 1 | 74 | 0.0 |
|  |  |  | 72S | 4194 | | 1223 | 1 | 29 | 0.1 |
|  | 14 | 12+0 | 0 E | 1017 | | 318 | 0 | 31 | 0.1 |
|  |  |  | 0S | 1344 | | 381 | 1 | 28 | 0.2 |
|  |  |  | 24E | 2842 | | 1491 | 0 | 52 | 0.0 |
|  |  |  | 24S | 676 | | 295 | 1 | 44 | 0.2 |
|  |  |  | 72E | 39637 | | 39637 | 1 | 100 | 0.0 |
|  |  |  | 72S | 1328 | | 381 | 0 | 29 | 0.0 |
|  | 15 | 12+1 | 0 E | 2384 | | 520 | 104 | 22 | 8.8 |
|  |  |  | 0S | 1747 | | 357 | 73 | 20 | 8.4 |
|  |  |  | 24E | 2974 | | 1413 | 90 | 48 | 6.1 |
|  |  |  | 24S | 1623 | | 349 | 63 | 22 | 7.8 |
|  |  |  | 72E | 24470 | | 18242 | 90 | 75 | 0.7 |
|  |  |  | 72S | 1732 | | 792 | 77 | 46 | 8.8 |
|  | 16 | 17+3 | 0 E | 1367 | | 412 | 0 | 30 | 0.0 |
|  |  |  | 0S | 1522 | | 419 | 1 | 28 | 0.1 |
|  |  |  | 24E | 1918 | | 474 | 0 | 25 | 0.0 |
|  |  |  | 24S | 1413 | | 311 | 0 | 22 | 0.0 |
|  |  |  | 72E | 6896 | | 5040 | 0 | 73 | 0.0 |
|  |  |  | 72S | 2081 | | 792 | 1 | 38 | 0.1 |
|  | 17 | 13+0 | 0 E | 4737 | | 738 | 1 | 16 | 0.0 |
|  |  |  | 0S | 3332 | | 660 | 0 | 20 | 0.0 |
|  |  |  | 24E | 7867 | | 3681 | 0 | 47 | 0.0 |
|  |  |  | 24S | 3060 | | 567 | 1 | 19 | 0.0 |
|  |  |  | 72E | 39637 | | 39637 | 0 | 100 | 0.0 |
|  |  |  | 72S | 3611 | | 808 | 1 | 22 | 0.1 |
|  | 18 | 15+4 | 0 E | 2831 | | 617 | 128 | 22 | 9.0 |
|  |  |  | 0S | 2097 | | 454 | 82 | 22 | 7.8 |
|  |  |  | 24E | 11754 | | 9226 | 119 | 78 | 2.0 |
|  |  |  | 24S | 2435 | | 687 | 83 | 28 | 6.9 |
|  |  |  | 72E | 59456 | | 59456 | 90 | 100 | 0.3 |
|  |  |  | 72S | 3355 | | 1340 | 78 | 40 | 4.6 |
|  | 19 | 12+6 | 0 E | 3902 | | 1072 | 0 | 27 | 0.0 |
|  |  |  | 0S | 2318 | | 676 | 0 | 29 | 0.0 |
|  |  |  | 24E | 4135 | | 1491 | 4 | 36 | 0.2 |
|  |  |  | 24S | 4100 | | 1153 | 0 | 28 | 0.0 |
|  |  |  | 72E | 12057 | | 7525 | 0 | 62 | 0.0 |
|  |  |  | 72S | 4263 | | 1211 | 0 | 28 | 0.0 |
|  | 20 | 13+2 | 0 E | 3809 | | 909 | 200 | 24 | 10.5 |
|  |  |  | 0S | 5056 | | 1107 | 238 | 22 | 9.4 |
|  |  |  | 24E | 10274 | | 6151 | 194 | 60 | 3.8 |
|  |  |  | 24S | 3448 | | 897 | 143 | 26 | 8.3 |
|  |  |  | 72E | 59456 | | 59456 | 180 | 100 | 0.6 |
|  |  |  | 72S | 6174 | | 1456 | 263 | 24 | 8.5 |

* This sample was not included in figure 1. ‘-‘ indicates that gestational age is unknown.
