# Supplementary material for: Implementing Prenatal Diagnosis Based on Cell-Free Fetal DNA: Accurate Identification of Factors Affecting Fetal DNA Yield
Source: PLoS One. 2011 Oct 4;6(10):e25202. doi: 10.1371/journal.pone.0025202 (PMC3187716; doi:10.1371/journal.pone.0025202)
Supplement: Table S2 — Summary statistics. (DOC) [file pone.0025202.s002.doc]

**Supplementary Table 2:** Summary Statistics

|  | **Time to Process (hrs)** | **Total Copies DNA/ml plasma** | **Long DNA Copies/ml plasma** | **Total Copies Male DNA/ml plasma** | **% Long DNA** | **% male DNA** |
| --- | --- | --- | --- | --- | --- | --- |
| Median | 0 | 4395 | 1228 | 147 | 26 | 10.3 |
|  | 4 | 4146 | 1033 | 151 | 26 | 9.1 |
|  | 24 | 6748 | 3541 | 196 | 50 | 6.2 |
| Average | 0 | 4478 | 1160 | 240 | 26 | 11.7 |
|  | 4 | 4728 | 1247 | 228 | 25 | 10.9 |
|  | 24 | 8512 | 4667 | 243 | 51 | 7.4 |
| Number of samples |  | 12 | 12 | 10 | 12 | 10 |
| 5^th^ percentile | 0 | 1783 | 428 | 117 | 21 | 6.1 |
|  | 4 | 1684 | 552 | 73 | 13 | 4.9 |
|  | 24 | 2291 | 951 | 67 | 38 | 3.0 |
| 95^th^ percentile | 0 | 8448 | 1905 | 622 | 33 | 22.3 |
|  | 4 | 10352 | 2607 | 616 | 34 | 21.1 |
|  | 24 | 17440 | 10916 | 683 | 69 | 14.9 |
| Module 3 |  |  |  |  |  |  |
| Median | 0 | 3359 | 897 | 107 | 24 | 7.5 |
|  | 8RT | 3949 | 1290 | 123 | 29 | 8.2 |
|  | 8 4C | 3569 | 1014 | 114 | 29 | 8.5 |
|  | 24 RT | 5491 | 2093 | 106 | 42 | 6.4 |
|  | 24 4C | 5141 | 2353 | 114 | 43 | 4.7 |
|  | 72 | 14833 | 11722 | 100 | 83 | 1.5 |
| Average | 0 | 3668 | 943 | 134 | 26 | 8.2 |
|  | 8RT | 4195 | 1322 | 151 | 31 | 8.2 |
|  | 8 4C | 3690 | 1080 | 124 | 30 | 8.2 |
|  | 24 RT | 5831 | 2870 | 148 | 47 | 6.0 |
|  | 24 4C | 4749 | 2169 | 134 | 45 | 6.1 |
|  | 72 | 16801 | 14127 | 113 | 77 | 2.2 |
| Number of samples |  | 10 | 10 | 6 | 10 | 6 |
| 5^th^ Percentile | 0 | 2316 | 431 | 72 | 17 | 5.1 |
|  | 8RT | 2270 | 639 | 74 | 23 | 3.8 |
|  | 8 4C | 2240 | 670 | 85 | 22 | 4.8 |
|  | 24 RT | 3155 | 1318 | 65 | 34 | 2.4 |
|  | 24 4C | 3488 | 959 | 62 | 35 | 3.4 |
|  | 72 | 7551 | 4228 | 42 | 53 | 0.3 |
| 95^th^ Percentile | 0 | 5793 | 1608 | 231 | 37 | 13.2 |
|  | 8RT | 6916 | 2152 | 275 | 45 | 12.9 |
|  | 8 4C | 5443 | 1634 | 189 | 38 | 11.5 |
|  | 24 RT | 10000 | 6180 | 295 | 66 | 9.8 |
|  | 24 4C | 6841 | 3152 | 243 | 62 | 10.4 |
|  | 72 | 33036 | 32232 | 201 | 97 | 4.9 |
| Module 4 |  |  |  |  |  |  |
| Median | 0E | 3460 | 692 | 139 | 26 | 7.4 |
|  | 0S | 2668 | 713 | 180 | 27 | 6.8 |
|  | 24E | 3730 | 1491 | 137 | 39 | 5.5 |
|  | 24S | 2885 | 691 | 143 | 26 | 6.2 |
|  | 72E | 14262 | 10317 | 136 | 75 | 1.5 |
|  | 72S | 3654 | 1149 | 162 | 29 | 6.3 |
| Average | 0E | 3479 | 843 | 153 | 25 | 7.6 |
|  | 0S | 3228 | 822 | 154 | 27 | 7.4 |
|  | 24E | 5129 | 2340 | 149 | 42 | 5.4 |
|  | 24S | 3061 | 788 | 126 | 27 | 6.9 |
|  | 72E | 23256 | 19942 | 147 | 79 | 1.5 |
|  | 72S | 3567 | 1037 | 153 | 31 | 6.8 |
| Number of samples |  | 20 | 20 | 9 | 20 | 9 |
| 5^th^ Percentile | 0E | 1350 | 407 | 98 | 16 | 5.5 |
|  | 0S | 1513 | 380 | 73 | 20 | 5.6 |
|  | 24E | 2169 | 784 | 94 | 26 | 2.7 |
|  | 24S | 1229 | 310 | 66 | 19 | 5.5 |
|  | 72E | 6709 | 4917 | 81 | 65 | 0.5 |
|  | 72S | 1324 | 425 | 73 | 22 | 4.8 |
| 95^th^ Percentile | 0E | 6211 | 1650 | 218 | 32 | 10.1 |
|  | 0S | 6209 | 1410 | 232 | 38 | 9.5 |
|  | 24E | 10348 | 6305 | 213 | 61 | 8.4 |
|  | 24S | 5603 | 1282 | 183 | 34 | 9.1 |
|  | 72E | 59456 | 59456 | 224 | 100 | 3.2 |
|  | 72S | 6684 | 1530 | 257 | 41 | 9.2 |
